# Free Trehalose Accumulation in Dormant *Mycobacterium smegmatis* Cells and Its Breakdown in Early Resuscitation Phase

**DOI:** 10.3389/fmicb.2017.00524

**Published:** 2017-03-30

**Authors:** Margarita O. Shleeva, Kseniya A. Trutneva, Galina R. Demina, Alexander I. Zinin, Galina M. Sorokoumova, Polina K. Laptinskaya, Ekaterina S. Shumkova, Arseny S. Kaprelyants

**Affiliations:** ^1^A.N. Bach Institute of Biochemistry, Federal Research Centre ‘Fundamentals of Biotechnology’ of the Russian Academy of SciencesMoscow, Russia; ^2^Zelinsky Institute of Organic Chemistry – Russian Academy of SciencesMoscow, Russia; ^3^Moscow Technological University, cluster MITHTMoscow, Russia

**Keywords:** dormant mycobacteria, *Mycobacterium smegmatis*, trehalose, trehalase, resuscitation

## Abstract

Under gradual acidification of growth medium resulting in the formation of dormant *Mycobacterium smegmatis*, a significant accumulation of free trehalose in dormant cells was observed. According to ^1^H- and ^13^C-NMR spectroscopy up to 64% of total organic substances in the dormant cell extract was represented by trehalose whilst the trehalose content in an extract of active cells taken from early stationary phase was not more than 15%. Trehalose biosynthesis during transition to the dormant state is provided by activation of genes involved in the OtsA-OtsB and TreY-TreZ pathways (according to RT-PCR). Varying the concentration of free trehalose in dormant cells by expression of *MSMEG_4535* coding for trehalase we found that cell viability depends on trehalose level: cells with a high amount of trehalose survive much better than cells with a low amount. Upon resuscitation of dormant *M. smegmatis*, a decrease of free trehalose and an increase in glucose concentration occurred in the early period of resuscitation (after 2 h). Evidently, breakdown of trehalose by trehalase takes place at this time as a transient increase in trehalase activity was observed between 1 and 3 h of resuscitation. Activation of trehalase was not due to *de novo* biosynthesis but because of self-activation of the enzyme from the inactive state in dormant cells. Because, even a low concentration of ATP (2 mM) prevents self-activation of trehalase *in vitro* and after activation the enzyme is still sensitive to ATP we suggest that the transient character of trehalase activation in cells is due to variation in intracellular ATP concentration found in the early resuscitation period. The negative influence of the trehalase inhibitor validamycin A on the resuscitation of dormant cells proves the importance of trehalase for resuscitation. These experiments demonstrate the significance of free trehalose accumulation for the maintenance of dormant mycobacterial viability and the involvement of trehalose breakdown in early events leading to cell reactivation similar to yeast and fungal spores.

## Introduction

The dormant state of infectious agents attracts attention from both microbiologists and physicians as their dormant forms are considered to be responsible for the development of chronic diseases characterized by phenotypic resistance to antibiotics. Thus, the transition of viable cells of *Mycobacterium tuberculosis* (MTB) to the dormant state causes latent TB – the asymptotic disease spread through a third of the human population (Lillebaek et al., 2002). Despite recent intensive studies of the molecular mechanisms underlining the transition of viable MTB cells to dormant forms (for review see, Dutta and Karakousis, 2014) much less is known about the mechanisms and metabolic processes responsible for dormant cells surviving for long periods and their resuscitation to a viable, multiplying state. It is known that bacterial cells accumulate some storage substances (polyphosphates, glycogen, PHB, etc.) which could be used to maintain metabolic activity and survival under nutrient-limiting conditions (Preiss, 1984; Wood and Clark, 1988; Anderson and Dawes, 1990).

Dormant bacterial spores contain a significant amount (up to 20%) of dipicolinic acid which is extremely important for spore resistance, stability and in protecting spore DNA from damage (Setlow et al., 2006). Yeast and fungal spores accumulate another storage/protective substance – trehalose – which participates in spore stabilization in stressful conditions like desiccation and could be used in spore germination (Elbein et al., 2003).

In a similar vein, we suggest that non-sporulating mycobacteria may accumulate in dormant cells some storage material which could contribute to both long-term persistence and resuscitation processes. The present study aimed to elucidate this possibility using dormant *Mycobacterium smegmatis* – a non-pathogenic *Mycobacterium* which is able to produce a dormant form similar to MTB.

## Materials and Methods

### Bacterial Strains, Growth Media, and Culture Conditions

*Mycobacterium smegmatis* mc^2^ 155 was initially grown for 24 h in Nutrient Broth (“Himedia”) in the presence of 0,05% Tween-80 at 37°C under agitation (220 rpm). The culture, grown in the above medium, served as an inoculum that was added to 250 ml of modified Sauton’s medium at a concentration 10^5^–10^6^ cells per ml, containing (per liter): KH_2_PO_4_, 0.5 g; MgSO_4_⋅7H_2_O, 1.4 g; l-asparagine, 4 g; glycerol, 60 ml; ferric ammonium citrate, 0.05 g; sodium citrate, 2 g; 1% ZnSO_4_⋅7H_2_O, 0.1 ml; H_2_O, to l L supplemented by 0,05% Tween-80. Modification of Sauton’s medium included initial pH reduced to pH 6.0–6.2 (no addition of NaOH) (Kudykina et al., 2011). Cultures were incubated in modified Sauton’s medium at 37°C with shaking for 10–20 days and pH values were periodically measured. When the medium in post-stationary phase *M. smegmatis* cultures reached pH 6.0–6.2 (after 13–15 days), cultures were transferred to plastic capped tubes (50 ml) and kept further under static conditions without agitation at room temperature for up to 100 days post-inoculation.

To produce samples enriched with dormant cells from stationary tube cultures cells were centrifuged (5000 rpm for 10 min) and washed by centrifugation 10 times with the buffer contained NaCl – 8 g, KCl – 0.2 g, Na_2_HPO_4_ – 0.24 g, H_2_O to 1000 ml, PH 7.4. The faction obtained was suspended in the reactivation medium. As the result of this procedure, a homogeneous fraction of dormant cells contained no less than 80% of resuscitable cells was obtained.

### Viability Estimation by CFU

Bacterial suspensions were serially diluted in fresh Sauton’s medium, and then three replicate 100 μl samples from each dilution were spotted on NBE agar. Plates were incubated at 37°C for 5 days then the number of colony forming units (CFUs) was counted. The limit of detection was 10 CFU/ml.

### Resuscitation of the Dormant Cells

The resuscitation of NC cells of *M. smegmatis* was accomplished using reactivation medium. This is twice diluted Sauton’s medium (Nikitushkin et al., 2013) supplemented with 0.6% glycerol and 0.025% yeast extract (LabM).

The resuscitation was carried out in two formats:

For most probable number (MPN) format, 48-well plastic plates (Corning, NY, USA) were used; each well-contained 1 ml reactivation medium. Some wells were supplemented with validamycin A at various concentrations. Serially diluted *M. smegmatis* NC cells were added to three replicate wells. Plates were incubated at 37°C with agitation at 100 rpm for 10–14 days and the number of wells with visible bacterial growth was scored. MPN values were determined using standard statistical tables (de Man, 1975).

For batch format, the *M. smegmatis* dormant cells, obtained as above after washing, were resuspended in 200 ml reactivation medium containing 0.025% tyloxapol in a 500 ml flask to give an initial OD_600_ = 0.3–0.4. Incubation was at 37°C for 24 h with agitation at 100–120 rpm and cultures were sampled periodically for the measurements. In some experiments samples were plated on NBE agar.

#### Extraction of Soluble Substances from Mycobacterial Cells

The soluble substances were extracted from the biomass based on the procedure described by Bligh and Dyer (1959). One milliliter of chloroform and 2 ml of methanol were added to 0.8 g of the wet biomass of the cells. Cells were agitated for 12 h in the extraction mixture with the subsequent centrifugation followed by the addition of the 1 ml of water and 1 ml of chloroform (to separate the phases). The water-methanol layer was analyzed by TLC, NMR, or HPLC.

#### NMR Analysis

Five ml of the water-methanol layer obtained after chloroform-methanol cell extraction (see above) containing about 10 mg/ml of organic substances were dried and dissolved in 1 ml of D_2_O. Spectra were recorded using a Bruker AM-300 spectrometer at 100 MHz.

#### Thin Layer Chromatography

Ten μl of the water-methanol layer was applied on silica gel 60 F_254_ contained plates (Sorbfil, Russia). Chromatography was carried out using solvent mixture 1-propanol:ethylacetate:water (6:1:3). Spot visualization was done with the mixture of 10% H_2_SO_4_ in ethanol followed by a thermal treatment.

#### HPLC Analysis of Trehalose and Glucose Concentrations

Ten μl of the water-methanol layer obtained after chloroform-methanol cell extraction (see above) was injected in Zorbax Carbohydrate column [size 4.6 mm × 150 mm (Agilent, USA)] and chromatographed at room temperature with Aquilon (Russia) HPLC chromatograph equipped with the refractometric detector. Mobile phase was acetonitrile:water (70:30), flow rate was 1 ml/min.

#### Metabolic Activity Estimation

The metabolic activity of cells was determined by monitoring of the incorporation of ^3^H-uracil. Samples of cell suspensions (1 ml) were incubated with 1 μl [5,6-^3^H] uracil (10 μCi; 0.2 μmol in 50% ethanol) and incubated for 4 h at 37°C with agitation (45–60 rpm). The cells were then harvested on glass fiber GFC filters (Whatman, UK), washed with 3 ml 7% trichloroacetic acid followed by washing with 3 ml absolute ethanol. Air-dried filters were placed in scintillation liquid and the radioactivity incorporated was measured with a scintillation counter LS6500 (Beckman, USA).

#### ATP Determination

Cells (1 ml) were collected within 8 h from the beginning of reactivation. After washing with phosphate buffer cells were destroyed by the beads homogenizer FastPrep-24 (MP Biomedicals, USA). Cell debris were separated by centrifugation and aliquots of 20 μl of supernatant were mixed with “ATP reagent” (100 μl) (Lumtek, Russia) and luminescence was measured by chemiluminometer Lum-5773 (Disoft, Russia). ATP standards diluted in PBS buffer were prepared fresh for each experiment.

#### RNA Isolation

RNA was extracted during transition of *M. smegmatis* cells to dormant state. For each time point, 30 ml culture samples were employed from three independent experiments. Cells were harvested by centrifugation (4000 *g*, 10 min) and 1 ml Trizol reagent was added to the pellets. Cells were disrupted using zirconia beads (0.1 mm) in the beads homogenizer FastPrep-24 (MP Biomedicals, USA). After centrifugation to remove particulates the supernatant was extracted once with chloroform. Nucleic acids were then precipitated with isopropanol, harvested by centrifugation, washed with 70% ethanol and re-dissolved in nuclease-free water (Promega, USA) containing RNAsin ribonuclease inhibitor (Promega, USA). RNA was then isolated using an RNeasy Mini kit (Qiagen). Each RNA sample was finally treated with RNase-free DNase1 (Ambion), which was then heat-inactivated according to the kit protocol. RNA was quantified using a Touch Duo Spectrophotometer (BioDrop, UK).

### Quantitative Real-Time PCR

One microgram of total RNA was used for cDNA synthesis with random hexanucleotides and SuperScript III reverse transcriptase (Life Technologies). qPCR was performed using qPCRmix-HS SYBR (Evrogen, Russia) and the LightCycler 480 Real-Time PCR system (Roche, Switzerland); cycling conditions were as follows: 95° for 20 s, 60° for 20 s, 72° for 30 s, repeat 40 times; gene-specific primers are listed in Supplementary Table S1. Amount of 16S rRNA in each sample was used as a reference.

#### Assay of Trehalase Activity

The enzymatic activity of the trehalase was measured by estimation of released glucose with the glucose oxidase test kit (Nelson, 1944). Assay mixtures contained the following components in a final volume of 100 μL: 100 mM phosphate buffer (pH 7.1), 6 mM MgCl_2_, 50 mM trehalose, and various amounts of cell lysate obtained as described for ATP determination (Carroll et al., 2007). Assay mixture was incubated at 37°C for 5–200 min.

#### pES Expression Vector Construction

pES tetracycline-inducible expression vector was constructed on the base of pKW08-Lx vector characterized by well-regulated tetracycline-dependent expression (Williams et al., 2010). Essentially, pMind-derived multiple cloning site (MCS) was cloned instead of *lux*-gene. The vector was constructed as follow. pMind polylinker was amplified with pMind-782F (5′-GCTACTCTCATCTGACTG-3′) and pMind-909R (5′-CAGGGAAGCTTGTGTCA-3′) primers (HindIII-site is underlined). The obtained PCR-product (144 bp) was digested with HindIII and BamHI enzymes. DNA fragment (90 bp) obtained was ligated into preliminary digested with the same restriction enzymes pKW08-Lx vector that led to the pES01 construction.

#### Construction of pES_MSMEG_4535 Expression Vector

Trehalase gene *MSMEG_4535* was amplified from the *M. smegmatis* mc^2^ 155 chromosomal DNA with F4535 (5′-TATCTGCAG*AAGAGGAGA*GCTGC**ATG**GTTCTGCAACAGACCGA-3′) and R4535 (5′-CACAAGCTTCGGCCAACAGCAGCCTCACC-3′) primers. The former included PstI-site (underlined), artificial ribosome-binding site (Italic) and the first 20 nucleotides of the gene (ATG start codon is shown in bold). The latter harbored HindIII site (underlined). The obtained PCR-product was TA-cloned into the pGEM-T easy vector (Promega, USA), digested with PstI and HindIII enzymes and subcloned into pES expression vector restricted with the same enzymes beforehand. The resultant pES01-4535 plasmid was finally transformed into the wild type mc^2^ 155 *M. smegmatis* strain by electroporation.

The enzymes used were purchased from Thermo Fisher Scientific (the former Fermentas, Lithuania). Plasmid DNA purification and DNA extraction from agarose gel were performed with Wizard Plus SV Minipreps DNA Purification System (Promega, USA) and Wizard SV Gel and PCR Clean-Up System (Promega, USA) respectively. pES and pES_MSMEG_4535 constructs were sequenced with pKW4f (5′-CGCTACTCTCATCGTGGAATC-3′) and pKW4r (5′-CCTCGAGGTCGACGGTAT-3′) primers. MSMEG_4535 gene insert in pGEM-T easy was checked and sequenced with standard M13-primers. The central region of MSMEG_4535 gene was sequenced with F4535seq (5′-AGGCCGACGACTTCTTCTC-3′) and R4535seq (5′-GGTGGTGCAGCTCTTCCTC-3′) primers.

All cloning procedures were performed in *Escherichia coli* BMH 71-18 mutS strain (Promega, USA). Recombinant gene expression was induced by 20 ng/ml Tetracycline hydrochloride (Acros Organics, USA).

## Results

### Free Trehalose Accumulation during Transition of *Mycobacterium smegmatis* Cells to a Dormant State

*Mycobacterium smegmatis* cells grown in Sauton medium under conditions of gradual acidification of the culture medium developed ovoid-shape dormant cells after 14 days of cultivation. The appearance of ovoid cells in the population was accompanied by a lowering of metabolic activity judged by a decrease of H^3^-uracil incorporation whilst the viability of cells (CFU) in the culture remained almost constant over 30 days (Kudykina et al., 2011).

**FIGURE 1:**
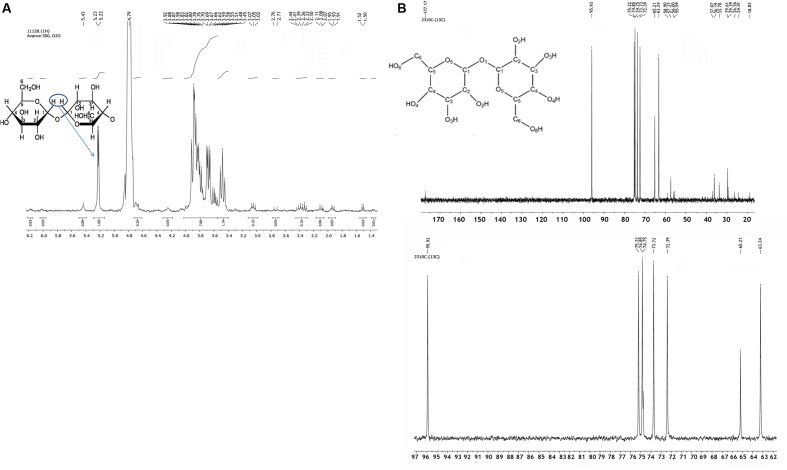
**^1^H-NMR** **(A)** and ^13^C-NMR **(B)** spectra of water-methanol fraction of chloroform- methanol extract of the dormant ovoid *Mycobacterium smegmatis* cells. Dormant cells were obtained after *M. smegmatis* cell growth in modified Sauton medium under gradual acidification (14 days of incubation) as described in M&M. Cells were centrifuged, washed and extracted by chloroform-methanol (for details see M&M). Duplet at 5.18–5.19 ppm in ^1^H-NMR **(A)** spectrum and peak at 95.96 ppm in ^13^C-NMR **(B)** belong to anomeric proton and to anomeric C-1 atom in trehalose molecule correspondently. For description of other peaks in each spectrum (see Results).

We analyzed the low molecular weight components of a water–methanol extract of dormant *M. smegmatis* cells (14 days of incubation) by ^1^H- and ^13^C-NMR. Comparison of the data obtained with published spectra of standard solutions of pure substances^1^ allowed identification of the main component of the cytoplasm of mycobacterial dormant cells as trehalose (anomeric proton – duplet at 5.18–5.19 ppm and complex of protons between 3.44 and 3.85 ppm in NMR spectrum; **Figure 1A**). The percentage of trehalose was estimated at about 64% of non-exchangeable protons with deuterium for dormant 14-day-old cells. The ^1^H-NMR spectrum of the water–methanol phase of the extract taken from actively growing mycobacterial cells also revealed the presence of trehalose in the cytoplasm of bacteria but in a much smaller amount (15% of total organic material; not shown). ^13^C-NMR confirmed trehalose accumulation in dormant cells. The signals of ^13^C-NMR chemical shifts (ppm) – 96, 75.2, 74.9, 73.8, 72.4, and 63.2 – were close to the published shifts for trehalose [anomeric C-1 atom (95.96 ppm), C-2 atom (75.215 ppm), C-3 atom (74.875 ppm), C-4 atom (73.765 ppm), C-5 atom (72.4 ppm), C-6 atom (63.246 ppm); **Figure 1B**].

**FIGURE 2:**
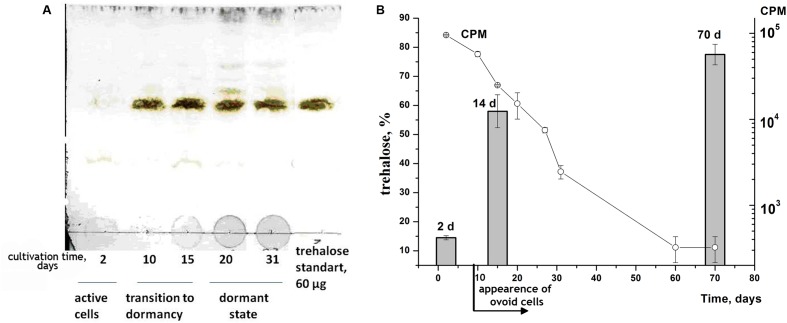
**Accumulation of trehalose during transition of *M. smegmatis* cells to dormancy.** *M. smegmatis* cells were cultivating under gradually acidification as described in M&M accompanied by accumulation of dormant ovoid cells. Periodically cell samples were withdrawn and water-methanol extracts were prepared. The extracts were analyzed by TLC **(A)** or by ^1^H-NMR **(B)**. Plates were stained by 10% H_2_SO_4_. Calculation of trehalose percentage on the basis ^1^H-NMR (closed bars, **B**) was done as describe in M&M. Incorporation of ^3^H-uracil in cells is represented as CPM in 0.2 ml of culture (open circles, **B**). Bars represent ± SD.

Water–methanol extracts of *M. smegmatis* cells taken from different physiological phases were chromatographed by TLC on a Merck RP-18 column. One major spot with Rf = 0.87 which corresponded to the position of standard trehalose was registered for dormant cells but was negligible in the extract from active cells (**Figure 2A**). The structure of the compound concentrated in major spots was confirmed as trehalose by ^1^H-NMR spectroscopy. Estimation of the trehalose content in cells by two methods (NMR and TLC) demonstrated its significant increase during the transition from active growth to dormancy which correlated with a reduction in the level of H^3^-uracil incorporation indicating the development of dormancy (**Figure 2B**). A high trehalose concentration was maintained over 2 months of cell storage (**Figure 2B**).

### Expression of Genes Involved in Trehalose Synthesis during Transition of *M. smegmatis* Cells to a Dormant State

It is known that trehalose is synthesized *de novo* in *M. smegmatis* via the OtsA-OtsB (from glucose-6-phosphate) and the TreY-TreZ (from cytosolic α-1,4-linked glucose polymers) pathways. Previously, suggested the third biosynthetic pathway for trehalose – TreS has been shown to contribute a little to trehalose biosynthesis in both *M. smegmatis* and *M. tuberculosis* (Miah et al., 2013; Kalscheuer and Koliwer-Brandl, 2014). In fact, TreS *in vitro* is able to synthesis trehalose from maltose but the opposite reaction is much faster (Murphy et al., 2005). In growing *M. smegmatis* cells the formation of trehalose from maltose via TreS is limited by biosynthesis of maltose (Miah et al., 2013). In order to establish the main root of trehalose synthesis during the formation of dormant *M. smegmatis* cells, expression of the genes involved in these reactions was investigated by real-time PCR. All studied genes were expressed in the middle exponential phase (3-day-old culture grown on Sauton medium with starting pH 6.0). RNA from this stage was taken as a positive calibrator for quantification of relative expression during transition of mycobacteria to the dormant state.

**FIGURE 3:**
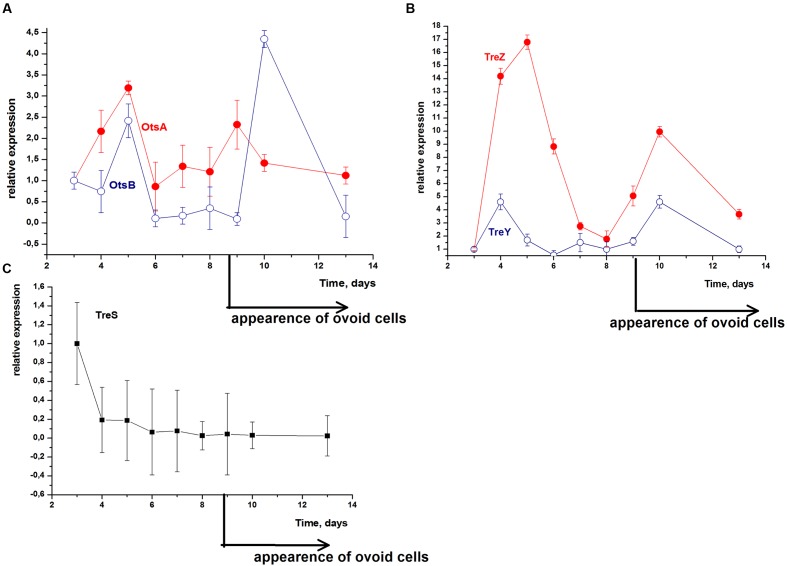
**Expression profiles of *M. smegmatis* genes responsible for different trehalose synthesis pathways during transition to dormancy.** *M. smegmatis* cells were grown as described in legend for **Figure 1**. RNA was isolated from cells withdrawn from the stationary culture at different time points. Quantitative RT-PCR was performed using cDNA obtained as described in Section “Materials and Methods.” Each point is the mean of three replicates. Error bars represent the standard error of the mean. Each point shows relative expression of particular gene (mRNA level) normalized to 16S RNA. mRNA level at time point “3 days” used for comparison. **(A)** Expression of key genes of OtsAB pathway, **(B)** expression of key genes of TreZY pathway, **(C)** expression of key genes of TreS pathway.

In the early stationary phase (ca. 4–5 days) an increase in the relative expression of almost all the genes studied occurred: the expression of trehalose-phosphate synthase (*MSMEG_5892*; OtsA) was increased threefold and the expression of trehalose-6-phosphate phosphatase (*MSMEG_3954*; OtsB) was raised twofold 5 days after inoculation of the bacteria (**Figure 3A**).

Expression of maltooligosyl-trehalose synthase (*MSMEG_4696*; TreY) was increased by 4.5 times and expression of maltooligosyl-trehalose trehalohydrolase (*MSMEG_3184*; TreZ) was significantly increased by 16.5 times 4 days after inoculation (**Figure 3B**).

A second burst of expression of OtsA, OtsB, TreY, and TreZ enzymes was observed in the late stationary phase (9–10 days) when transition of the mycobacteria to the dormant state started (**Figures 3A,B**).

Gene expression of TreS enzyme was extremely low over the whole time period (**Figure 3C**).

In the dormant state (age of bacteria 13 days or more) the expression level of all genes of trehalose metabolism was significantly reduced (**Figure 3**).

Thus, the expression of genes responsible of all three pathways of trehalose synthesis increased during active growth (maximum at 4–5 days) whilst expression of OtsAB and TreYZ revealed a second maximum corresponding to the start of ovoid cell formation in response to significant acidification of the medium (9–11 days). Evidently, these two biosynthetic pathways (but not the degradatory pathway) are responsible for the accumulation of trehalose found in dormant cells.

### Survival of Dormant Cells Depends on Intracellular Trehalose Level and Trehalase Activity

**FIGURE 4:**
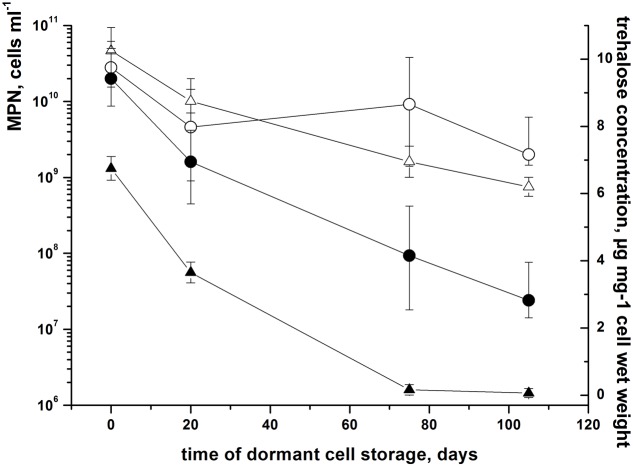
**Viability of dormant *M. smegmatis* cells correlates with intracellular trehalose content.** Dormant *M. smegmatis* cells contained empty pES plasmid (open symbols) and cells with over-expressed trehalase MSMEG_4535 (closed symbols) were obtained and kept under static conditions as describe in M&M. Periodically samples were withdrawn for trehalose level determination (triangles) and viability estimation by MPN assay (circles). Zero time corresponds to the time of transferring of 13–15 days old post-stationary phase cells to plastic capped tubes. This experiment was performed in three biological repeats. A typical result is shown. Bars represent (95%) confidence limits for the MPN assay. Bars for trehalose content represent ± SD.

In order to establish whether accumulated trehalose in dormant *M. smegmatis* cells plays a role in the maintenance of cell viability we checked cell viability with different levels of intracellular trehalose. Because the genes responsible for trehalose biosynthesis are essential (Woodruff et al., 2004) we changed the level of trehalose by modification of trehalase activity by the following approach: we over-expressed the trehalase gene *MSMEG_4535* in *M. smegmatis* (strain pES_MSMEG_4535) which resulted in an increase of specific trehalase activity in growing bacteria up to 10 times (Supplementary Figure S1). pES_MSMEG_4535 strain grew similar to control strain containing similar amount of trehalose in active growth phase and was able to form dormant cells. However, the intracellular concentration of trehalose in genetically modified dormant cells gradually decreased over the cell storage period and after 75 days was negligible (**Figure 4**). Estimation of viability of dormant cells by CFU and MPN assay revealed a direct correlation between trehalose content and cell viability (**Figure 4**). This mirrored the rise in the proportion of damaged cells in a low-trehalose cell population according to propidium iodide staining (not shown).

These results clearly demonstrate a link between trehalose content and maintenance of dormant cell viability for a long time without multiplication.

### Trehalose Content and Trehalase Activity Changes during Resuscitation of Dormant Bacteria

Dormant *M. smegmatis* cells are able to resume their multiplication in fresh medium after some period of ‘metabolic resuscitation’ which precedes cell division (Shleeva et al., 2013).

**FIGURE 5:**
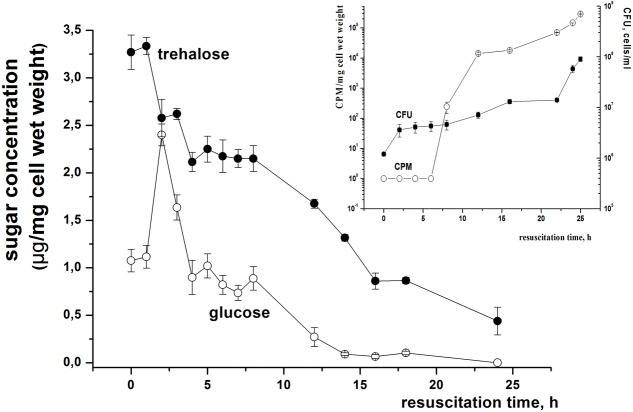
**Changes in trehalose and glucose level in *M. smegmatis* cells during reactivation from dormant state.** Dormant cells were obtained after 3.5–4.5 months of storage under static conditions as described in M&M. Cells were washed, inoculated in fresh Sauton medium and incubated under aeration at 37°C. Periodically samples were withdrawn for estimation of trehalose and glucose concentrations; CFU and H^3^-uracyl incorporation are shown in the insert. This experiment was repeated three times. One typical experiment is shown. Bars represent ± SD.

We found that intracellular trehalose content decreased in the initial period of resuscitation (1–5 h) – well before metabolic activity registered by uracil accumulation (8–12 h). The trehalose amount continued to drop until pronounced cell division (measured by CFU) started (after ca. 24 h) (**Figure 5**), approaching its intracellular level for viable log-phase cells (0.2–0.5 μg/mg cell wet weight after 48 h of cultivation). Evidently, the decrease of trehalose content is due to its hydrolysis as a transient increase in glucose concentration was recorded in the initial resuscitation phase (2 h) (**Figure 5**). Similarly to trehalose, glucose concentration dropped after 5 h of resuscitation until the level was close to log-phase multiplying cells (ca. 0.2 μg/mg cell wet weight). Probably, the decrease of glucose content could reflect glucose utilization by activated cellular metabolic pathways.

**FIGURE 6:**
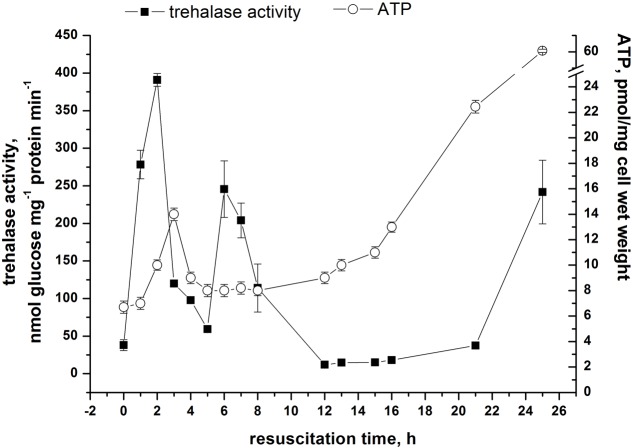
**Trehalase activity and ATP level in *M. smegmatis* cells during reactivation from dormant state.** Reactivation of dormant cells were performed similar to experiment shown on **Figure 4**. This experiment was repeated three times. One typical experiment is shown. Bars represent ± SD.

Because, trehalose hydrolysis is controlled by trehalase we studied trehalase activity in the resuscitation phase. This experiment revealed a significant transient increase of trehalase activity 2 h after the onset of resuscitation (**Figure 6**) which correlated with a burst of glucose and a rapid decrease in trehalose content (**Figure 5**). A second transient increase in trehalase activity was found 5–7 h after the start of reactivation. A third increase in trehalase activity was observed after 24 h when multiplication of cells started (**Figure 6**). This activity was maintained at an approximately constant level during log phase (not shown).

In order to uncover the reasons for fluctuating trehalase activity in the resuscitation phase, we studied the activity of trehalase in crude extract of viable and dormant cells *in vitro*.

**FIGURE 7:**
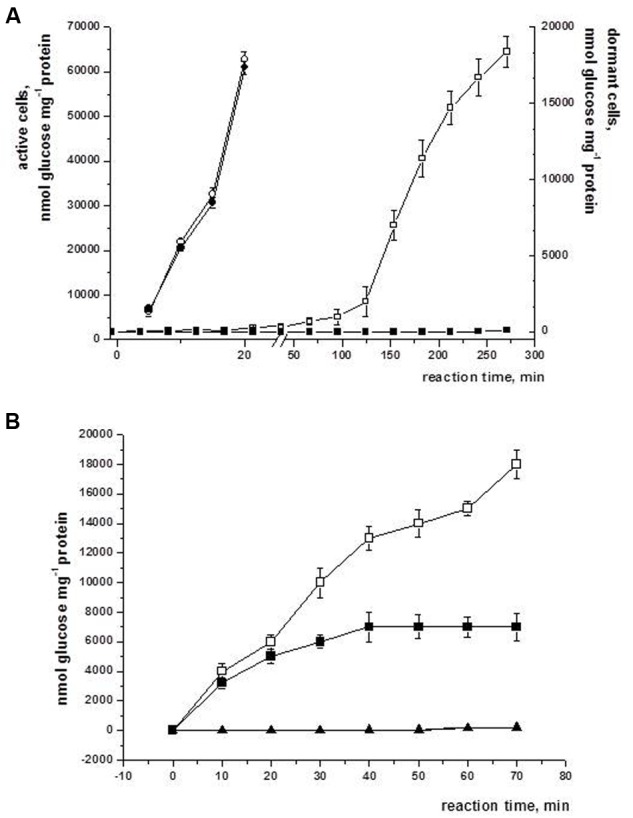
**Self-activation of trehalase isolated from dormant *M. smegmatis* cells *in vitro*.** Dormant cells of *M. smegmatis* were obtained after 3–4 months storage under static conditions as described in M&M. Active (2 days of growth in standard Sauton medium) and dormant cells were homogenized to obtain crude cytosolic fraction. Trehalase activity was determined in 100 mM phosphate buffer (for details see M&M). Circles – trehalase activity of active cells, squares – trehalase activity of dormant cells; open symbols – without ATP, closed symbols – with 2 mM ATP added to the reaction medium. **(A)** The reaction was carried out immediately after fraction obtained. **(B)** The cytosolic fraction was pre-incubated for 3 h at room temperature without (circles) or in the presence of 2 mM ATP (triangles). This experiment was repeated five times. One typical experiment is shown. Bars represent ± SD.

It is interesting that whilst trehalase activity (measured by accumulation of glucose) in viable cells was detected immediately after substrate addition, this activity for dormant cells exhibited a significant lag phase before trehalose degradation started, demonstrating self-activation of the enzyme *in vitro* (**Figure 7A**). Indeed, pre-incubation of dormant cell extract for 3 h at room temperature removed the lag phase in the reaction after substrate addition (**Figure 7B**).

According to Carroll et al. (2007) trehalase activity in *M. smegmatis* is inhibited in the presence of 20 mM ATP. We checked the influence of ATP at a broad range of concentrations and found that activity of trehalase from dormant cells is not measurable in the presence of 2 mM ATP (**Figure 7B**). In contrast, trehalase of active cells was tolerant to this concentration of ATP and was inhibited only in the presence of more than 20 mM ATP (not shown). Interestingly, even after activation for 3 h the enzyme was partially sensitive to 2 mM ATP in the reaction mixture (**Figure 7B**). Evidently, 2 mM ATP completely arrests self-activation of trehalase from dormant cells (**Figure 7B**) resulting in an inactive enzyme (**Figure 7A**).

Thus, the observed fluctuation of trehalase activity in the course of resuscitation of dormant *M. smegmatis* cells (**Figure 6**) could be explained by the presence of trehalase in a latent (inactive) state in dormant cells and sensitivity of its activity to fluctuating ATP intracellular concentration. Indeed, as shown in (**Figure 6**) fluctuations of trehalase activity and ATP content are inversely correlated after 2 h when intracellular ATP concentration could reach threshold level (**Figure 6**). After 24 h of reactivation when active growth started high trehalase activity was detected under high concentration of ATP (**Figure 6**) which is in line with observed tolerance of trehalase to ATP in active cells.

The changes found in trehalose level and trehalase activity during reactivation of dormant *M. smegmatis* cells could suggest the significance of trehalose breakdown for the early resuscitation phase (0–12 h). To clarify, if trehalase from dormant cells is important for their reactivation we apply validamycin A (VM-A) – specific inhibitor of trehalases of different origins including bacteria (Li et al., 2012). At first we found that VM-A inhibited *M. smegmatis* trehalase *in vitro* in concentration above 1 μM (Supplementary Figure S2).

**FIGURE 8:**
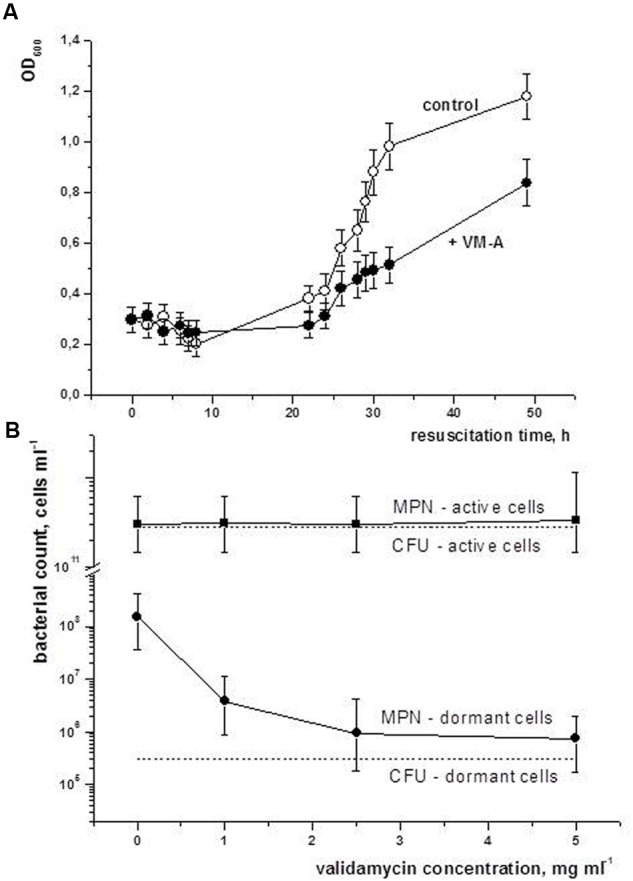
**Inhibition of trehalase activity influences reactivation of dormant *M. smegmatis* cells.** Dormant cells of *M. smegmatis* were obtained after 8–15 months storage under static conditions as described in M&M. **(A)** Reactivation in batch format was performed as describe in the legend for **Figure 4** with addition of the trehalase inhibitor VM-A. **(B)** Concentration dependence of VM-A influence on the number of viable cells after 10 days of reactivation. Reactivation was carried out in MPN format as describe in M&M. Different concentrations of VM-A were added to the each well with serially diluted resuscitating bacteria. Dash lines indicate CFU number for particular culture in the beginning of the experiment. The experiment **(A)** was repeated four times. The experiment **(B)** was repeated two times. Bars represent (95%) confidence limits for the MPN assay.

We checked the influence of VM-A on the resuscitation of dormant *M. smegmatis* cells. According to **Figure 8A** administration of VM-A to the resuscitation medium did not completely abolish cell reactivation but made the lag phase more prolonged and suppressed cell multiplication in the exponential phase (**Figure 8A**). In fact, the dormant cell population contained two types of cells: culturable (which can be estimated by CFU) and ‘non-culturable’ (NC) (cells which are unable to grow on solid medium but are able to multiply in liquid medium and can be estimated by MPN assay). The difference between the two parameters makes it possible to estimate the proportion of NC cells in the total dormant cell population (Shleeva et al., 2004) and study the influence of VM-A for the two populations separately. For 1.4-year-old dormant *M. smegmatis* cells the MPN/CFU difference approached 10^2^–10^3^ which means that in the population of dormant bacteria 99–99.9% of cells are NC. According to **Figure 8B** an increase of VM-A concentration in the resuscitation medium decreased the number of resuscitable NC cells (MPN number approaches CFU number at a VM-A concentration of 5 mg/ml in the case of dormant cells). Such a concentration of VM-A did not influence the MPN for viable (early stationary phase) cells which were, expectedly, almost the same as the CFU number (**Figure 8B**). Thus, the biological role of trehalose breakdown for the resuscitation of dormant culture is evidently vital for NC dormant *M. smegmatis* cells. Accordingly, the culturable cell sub-population could be responsible for the observed growth of dormant *M. smegmatis* cells in the presence of VM-A (**Figure 8A**).

Because VM-A also inhibits TreS enzyme (Kalscheuer et al., 2010), intracellular maltose level was checked over resuscitation period. It was found that during the first 24 h of resuscitation maltose concentration was maintained under constant and similar level in both cultures – untreated control and VM-A treated cells (Supplementary Figure S3). However, intracellular glucose level dropped in the initial stage of resuscitation (2–6 h) in the presence of VM-A in contrast to control culture where glucose level increased at the same time gap which reflects inhibition of trehalase by VM-A and possible consumption of initially available glucose. This experiment demonstrates that TreS activity is not involved in early stage of resuscitation and VM-A effects are solely attributed by inhibition of trehalase activity at least in resuscitation phase.

## Discussion

This study describes for the first time the accumulation of free trehalose in significant amounts by dormant mycobacterial cells. It is interesting that two known interchangeable *in vitro* pathways of trehalose biosynthesis (OtsAB and TreYZ) (Miah et al., 2013) participate in trehalose synthesis upon transition from the active to the dormant state according to RT-PCR (the second burst of gene transcription; **Figure 3**). In the mouse model of active tuberculosis the OtsAB2 pathway, which generates trehalose from glucose and glucose-6-phosphate, is the dominant pathway required for *M. tuberculosis* growth and virulence (Murphy et al., 2005). However, recent studies revealed that essentiality of *OtsB2* gene *in vivo* for the acute infection is due to self-poisoning of knock-outed cells by accumulation of trehalose-6-phosphate (Korte et al., 2016). Inactivation of the second (TreYZ) pathway, which generates trehalose from α-1,4-linked glucose polymers, had no effect on the growth of *M. tuberculosis* in mice (Murphy et al., 2005). Taking together these results suggest interchangeability of two pathways *in vivo* as well. Whilst TreS enzyme is reversible, it acts toward formation of maltose from trehalose in growing *M. smegmatis* (Miah et al., 2013) participating in novel pathway from trehalose to α-glucan (Kalscheuer et al., 2010; Koliwer-Brandl et al., 2016). However, we cannot exclude activity of TreS in opposite direction upon development of dormancy. TreS protein was found in the dormant cell proteome (Trutneva, personal communication) and therefore could be functional.

Trehalose in mycobacteria plays an important role as part of the complex of cell wall molecules (in the form of trehalose mycolate, ‘cord factor’) which determines the pathogenicity and virulence of the infectious agent of tuberculosis, leprosy, and some other diseases (Kalscheuer and Koliwer-Brandl, 2014). At the same time, being non-reducing, trehalose possesses several unique properties including high hydrophilicity, chemical stability, non-hygroscopic glass formation and no internal hydrogen bond formation (Romeo, 2012). The combination of these features explains the principal role of trehalose as a stress metabolite (Strøm and Kaasen, 1993). Indeed, a number of prokaryotic species respond to environmental stress by accumulation of cytoplasmic trehalose, either by transport of trehalose or by endogenous synthesis. Trehalose has been shown to protect *E. coli* (Strøm and Kaasen, 1993), *Corynebacterium glutamicum* (Wolf et al., 2003) and various cyanobacteria (Kempf and Bremer, 1998) during osmotic stress where it acts as a compatible solute. It is important for both cold tolerance (Kandror et al., 2002) and thermal tolerance (Hengge-Aronis et al., 1991) in *E. coli*. Trehalose protects yeast during thermal stress (Singer and Lindquist, 1998b), desiccation (Singer and Lindquist, 1998a), and oxidative stress (Benaroudj et al., 2001). In a previously published report on *M. smegmatis* it has also been shown that trehalose confers protection during elevated temperature (Woodruff et al., 2004). Trehalose is also an effective protectant of biological macromolecules including DNA (Woodruff et al., 2004; Bezrukavnikov et al., 2014). Trehalose prevents denaturation and aggregation of damaged proteins and thus facilitates their repair at a later stage (Strøm and Kaasen, 1993). All these effects could contribute to the structural stability of dormant cells during long persistence without division and low metabolic activity. The free trehalose in dormant *M. smegmatis* cells observed in this study is reminiscent of the accumulation of trehalose in large amounts in dormant yeast ascospores, actinomycete spores, and in fungal spores (Arguelles, 2000; Elbein et al., 2003). As suggested, trehalose decreases the mobility of cytoplasmic water resulting in global down-regulation of enzyme activity and spore polymer stabilization providing their prolonged viability (Van Laere et al., 1987). Another possible role of trehalose in dormant mycobacteria might be its usage as a carbohydrate store as proposed for yeast and filamentous fungal spores (Thevelein, 1984). Indeed, in yeast spores trehalose could be very slowly catabolized producing glucose and eventually ATP to maintain spore viability (Barton et al., 1982; Thevelein et al., 1982). We found that even after 4 months of storage dormant *M. smegmatis* contained some level of ATP (**Figure 6**) and glucose (**Figure 5**). Whilst, a possible link between the breakdown of trehalose and the level of ATP/glucose in dormant *M. smegmatis* is not evident we may speculate that upon a decrease of intracellular ATP concentration to a point lower than some critical level (less than 2 mM) trehalose breakdown may occur followed by formation of free glucose for usage in glycolytic reactions and ATP production. Indeed, all key enzymes of the glycolytic pathway in *M. smegmatis* were found in the proteome of dormant cells (Trutneva, personal communication). Such a feedback mechanism could economically control the energetic demands of dormant cells under persistent conditions. Of course, under our experimental conditions (high glycerol concentration in the surrounding dormant cells medium) breakdown of trehalose as carbon storage for maintaining of cell viability is questionable. However, the mechanism of slow remobilizing trehalose could be important under starvation conditions (Kalscheuer and Koliwer-Brandl, 2014), which dormant mycobacteria could be experienced in nature or in a host organism. Therefore, we consider trehalose significance for dormant cells under particular conditions mainly as a protective and global regulatory substance.

Nevertheless, the significance of accumulated free trehalose in dormant cells for the maintenance of viability and integrity is clear from experiments in which the content of trehalose varied (**Figure 4**).

Similar to germination of yeast or actinomycetes we found a decrease in trehalose content during resuscitation of dormant *M. smegmatis* cells (**Figure 5**) which possibly was due to activation of trehalase earlier in the resuscitation process (Hey-Ferguson et al., 1973). Indeed, similar to spore germination, the trehalase activity of *M. smegmatis* demonstrates transient activation at 2 h of resuscitation which is followed by its rapid decrease (Thevelein et al., 1982; Thevelein and Jones, 1983). Evidently, the activation of trehalose is not due to protein synthesis *de novo* because the onset of biosynthetic activity of dormant *M. smegmatis* was registered much later (12–16 h) in the resuscitation period. Moreover, trehalase activation occurred even in cell free extract after 2 h when protein biosynthesis was evidently absent (**Figure 7A**). Thus, trehalase activity is likely masked in dormant cells as was proposed for yeast ascospores (Thevelein et al., 1982). Two possible mechanisms for this phenomenon were suggested: (1) trehalase activity in dormant cells is modulated by low molecular weight compounds [ATP-dependent inhibition (Thevelein et al., 1982), or activated by phosphorylation (Ortiz et al., 1983)]; (2) a low level of hydration in spores may account for the low activity of trehalase whilst an increased level of hydration activates the enzyme (McBride and Ensign, 1990).

Modulation of trehalase activity by ATP concentration seems plausible because trehalase in free cell extract from dormant mycobacteria revealed inhibition even by a low ATP concentration (**Figure 7A**). We cannot exclude also the role of hydration of ovoid *M. smegmatis* cells in trehalase activation as the initial step of reactivation, which could result in a decrease in ATP concentration followed by trehalase activation. The nature of the difference between sensitivity to ATP of the enzyme isolated from dormant and active cells is not clear; however, the presence of trehalase in large aggregates in dormant cells in contrast to active cells (unpublished observations) could modulate trehalase sensitivity to ATP.

Activation of trehalase upon resuscitation of dormant *M. smegmatis* (**Figure 6**) is accompanied by a decrease in trehalose content and an increase in glucose concentration (**Figure 5**) which may indicate utilization of released glucose in the initial period of resuscitation when complete metabolic machinery is not yet functional. Even though this possibility for yeast spores has not been experimentally proven (Barton et al., 1982) the retardation of reactivation found in the presence of the trehalase inhibitor VM-A (in contrast to its influence on the growth of active cells – unpublished observation) and decrease of initially available glucose under inhibition of trehalase/TreS (Supplementary Figure S3) could add some value to this hypothesis.

## Conclusion

This work demonstrates the significance of accumulation of trehalose for the formation and maintenance of the dormant state in non-sporulating mycobacteria and exit from this state. The role of trehalose accumulation in the long viability of yeast and fungal spores and trehalose breakdown for exit from dormancy and spore germination was intensively studied in the 1980s. We found a striking similarity between the results obtained in those studies and the results of the present study of dormant mycobacteria, revealing common features of dormant forms of non-sporulating bacteria and true spores.

A more detailed study of this aspect of dormancy could lead to an understanding of how mycobacteria maintain viability for long time in a dormant state which would be useful in the future for developing new compounds against latent tuberculosis.

## Author Contributions

AK and MS conceived and designed the experiments. MS, KT, GD, GS, and ES performed the experiments. AK and MS analyzed the data. AZ and PL contributed analysis tools. MS prepared figures and graphs. AK and MS wrote the manuscript. All the authors read and approved the final manuscript. AK and MS revised the manuscript.

## Conflict of Interest Statement

The authors declare that the research was conducted in the absence of any commercial or financial relationships that could be construed as a potential conflict of interest.

## Footnotes

1 http://www.bmrb.wisc.edu/metabolomics/mol_summary/show_data.php?molName=D_trehalose\&id=bmse000125\&whichTab=1
