# Supplementary material for: Free Trehalose Accumulation in Dormant Mycobacterium smegmatis Cells and Its Breakdown in Early Resuscitation Phase
Source: Front Microbiol. 2017 Mar 30;8:524. doi: 10.3389/fmicb.2017.00524 (PMC5371599; doi:10.3389/fmicb.2017.00524)
Supplement: Supplementary file 1 [file Table_1.PDF]

Supplementary Table S1.

Primers used for quantitative real-time PCR.

MSMEG_5892F (5'– TCGTCAAACCCGAGTACCAC –3')
MSMEG_5892R (5'- ATGTGCAGGAAGAAGCCGAT -3')
MSMEG_3954F (5'– ATCGGCATGAGTACGGGAAC – 3')
MSMEG_3954R (5'– TCTCTGGGATCTGCTTCGGA – 3')
MSMEG_4696F (5'– CACGACATCCACCGCTTCAT – 3')
MSMEG_4696R (5'– TAATCCGCCTTCACCAGACG – 3')
MSMEG_3184F (5'– TACCAGAGCACCCCGTCATA –3')
MSMEG_3184R (5'- CACATCGGGACCTTCACTCC -3')
MSMEG_3186F (5'– CAGTTCCGAATCCTGGCAGT – 3')
MSMEG_3186R (5'– CAAGCAGATGCGCAACATCA – 3')
MSMEG_6515F (5'– TGAAGAAGCGGTGCCAGTAG – 3')
MSMEG_6515R (5'– GGCGACTTCTACGTCTGGAG – 3')
