# Supplementary figures and images for: Free Trehalose Accumulation in Dormant Mycobacterium smegmatis Cells and Its Breakdown in Early Resuscitation Phase
Source: Front Microbiol. 2017 Mar 30;8:524. doi: 10.3389/fmicb.2017.00524 (PMC5371599; doi:10.3389/fmicb.2017.00524)

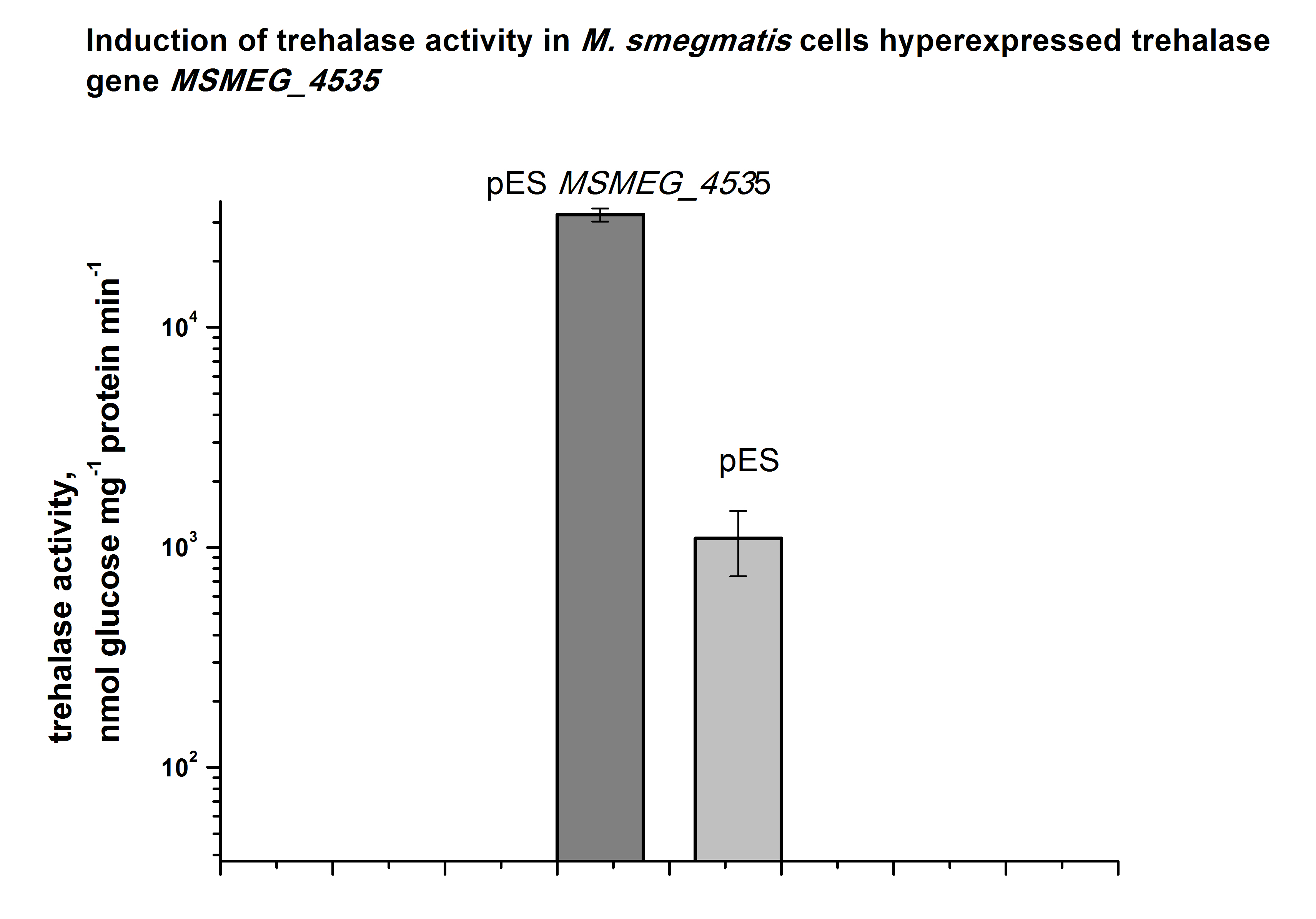

Supplement: Supplementary file 2 [file Image_1.JPEG]

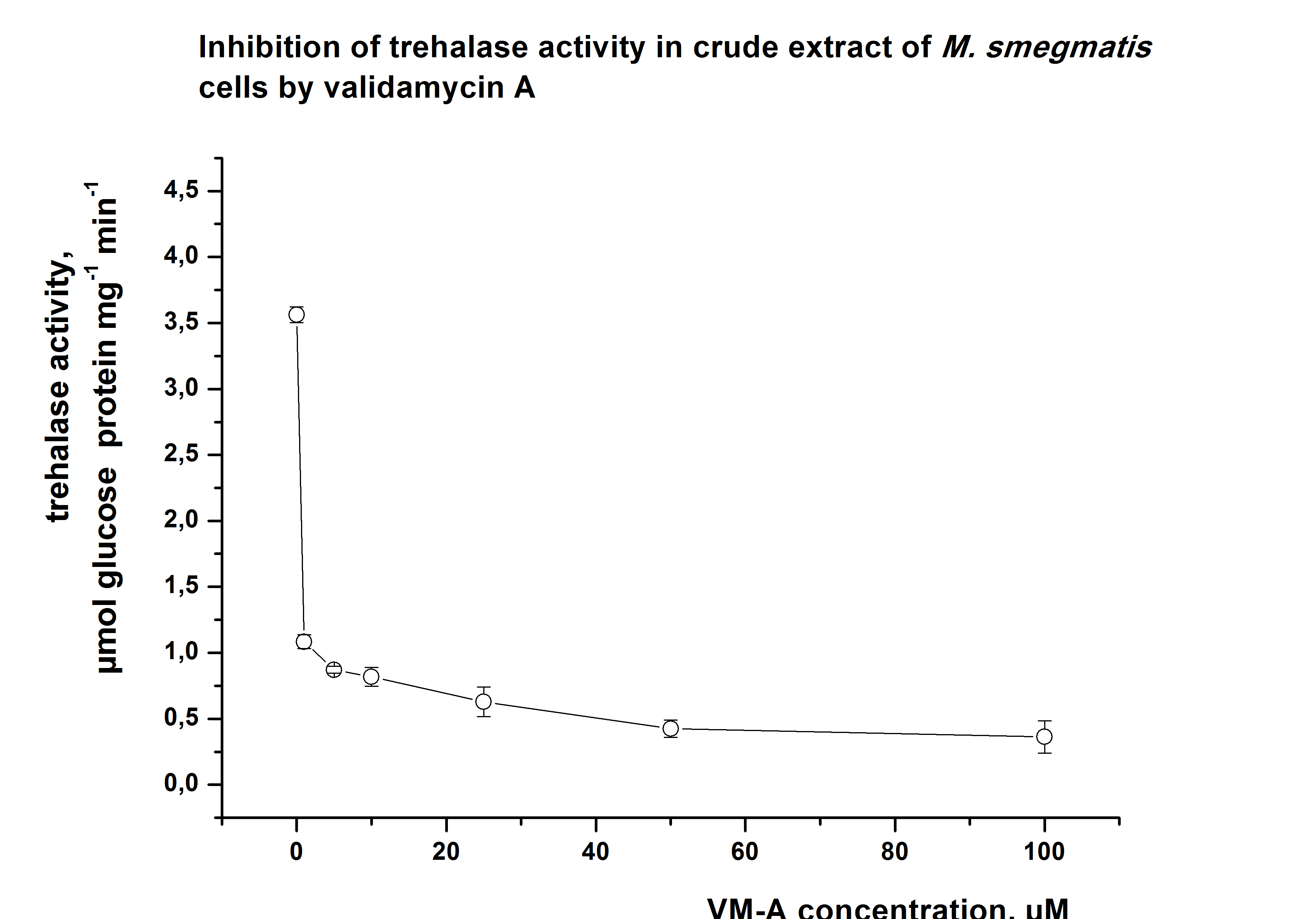

Supplement: Supplementary file 3 [file Image_2.JPEG]

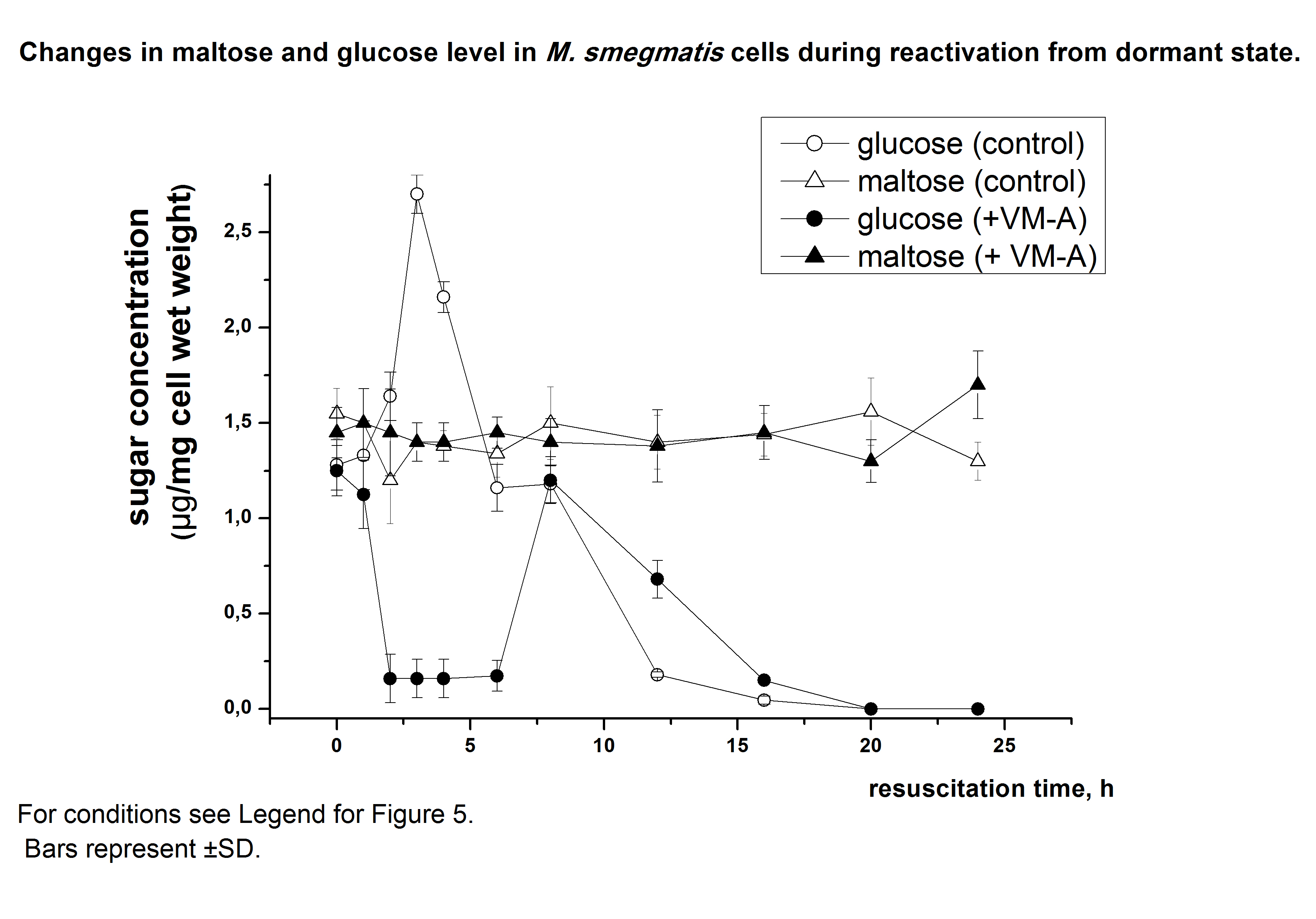

Supplement: Supplementary file 4 [file Image_3.JPEG]
